# Cause-specific childhood mortality in Africa and Asia: evidence from INDEPTH health and demographic surveillance system sites

**DOI:** 10.3402/gha.v7.25363

**Published:** 2014-10-29

**Authors:** P. Kim Streatfield, Wasif A. Khan, Abbas Bhuiya, Syed M.A. Hanifi, Nurul Alam, Mamadou Ouattara, Aboubakary Sanou, Ali Sié, Bruno Lankoandé, Abdramane B. Soura, Bassirou Bonfoh, Fabienne Jaeger, Eliezer K. Ngoran, Juerg Utzinger, Loko Abreha, Yohannes A. Melaku, Berhe Weldearegawi, Akosua Ansah, Abraham Hodgson, Abraham Oduro, Paul Welaga, Margaret Gyapong, Clement T. Narh, Solomon A. Narh-Bana, Shashi Kant, Puneet Misra, Sanjay K. Rai, Evasius Bauni, George Mochamah, Carolyne Ndila, Thomas N. Williams, Mary J. Hamel, Emmanuel Ngulukyo, Frank O. Odhiambo, Maquins Sewe, Donatien Beguy, Alex Ezeh, Samuel Oti, Aldiouma Diallo, Laetitia Douillot, Cheikh Sokhna, Valérie Delaunay, Mark A. Collinson, Chodziwadziwa W. Kabudula, Kathleen Kahn, Kobus Herbst, Joël Mossong, Nguyen T.K. Chuc, Martin Bangha, Osman A. Sankoh, Peter Byass

**Affiliations:** 1 Matlab HDSS, Bangladesh; 2 International Centre for Diarrhoeal Disease Research, Bangladesh; 3 INDEPTH Network, Accra, Ghana; 4 Bandarban HDSS, Bangladesh; 5 Chakaria HDSS, Bangladesh; 6 Centre for Equity and Health Systems, International Centre for Diarrhoeal Disease Research, Bangladesh; 7 AMK HDSS, Bangladesh; 8 Centre for Population, Urbanisation and Climate Change, International Centre for Diarrhoeal Disease Research, Bangladesh; 9 Nouna HDSS, Burkina Faso; 10 Nouna Health Research Centre, Nouna, Burkina Faso; 11 Ouagadougou HDSS, Burkina Faso; 12 Institut Supérieur des Sciences de la Population, Université de Ouagadougou, Burkina Faso; 13 Taabo HDSS, Côte d'Ivoire; 14 Centre Suisse de Recherches Scientifiques en Côte d'Ivoire, Abidjan, Côte d'Ivoire; 15 Swiss Tropical and Public Health Institute, Basel, Switzerland; 16 Université Félix Houphoët-Boigny, Abidjan, Côte d'Ivoire; 17 Kilite-Awlaelo HDSS, Ethiopia; 18 Department of Paediatrics and Child Health, College of Health Sciences, Mekelle University, Mekelle, Ethiopia; 19 Department of Public Health, College of Health Sciences, Mekelle University, Mekelle, Ethiopia; 20 Navrongo HDSS, Ghana; 21 Navrongo Health Research Centre, Navrongo, Ghana; 22 Dodowa HDSS, Ghana; 23 Dodowa Health Research Centre, Dodowa, Ghana; 24 Ballabgarh HDSS, India; 25 All India Institute of Medical Sciences, New Delhi, India; 26 Kilifi HDSS, Kenya; 27 KEMRI-Wellcome Trust Research Programme, Kilifi, Kenya; 28 Department of Medicine, Imperial College, St. Mary's Hospital, London; 29 Kisumu HDSS, Kenya; 30 KEMRI/CDC Research and Public Health Collaboration and KEMRI Center for Global Health Research, Kisumu, Kenya; 31 Nairobi HDSS, Kenya; 32 African Population and Health Research Center, Nairobi, Kenya; 33 Niakhar HDSS, Senegal; 34 Institut de Recherche pour le Developpement (IRD), Dakar, Sénégal; 35 Agincourt HDSS, South Africa; 36 MRC/Wits Rural Public Health and Health Transitions Research Unit (Agincourt), School of Public Health, Faculty of Health Sciences, University of the Witwatersrand, Johannesburg, South Africa; 37 Umeå Centre for Global Health Research, Umeå University, Umeå, Sweden; 38 Africa Centre HDSS, South Africa; 39 Africa Centre for Health and Population Studies, University of KwaZulu-Natal, Somkhele, KwaZulu-Natal, South Africa; 40 National Health Laboratory, Surveillance & Epidemiology of Infectious Diseases, Dudelange, Luxembourg; 41 FilaBavi HDSS, Vietnam; 42 Health System Research, Hanoi Medical University, Hanoi, Vietnam; 43 Public Health, Faculty of Health Sciences, University of the Witwatersrand, Johannesburg, South Africa; 44 Hanoi Medical University, Hanoi, Vietnam; 45 WHO Collaborating Centre for Verbal Autopsy, Umeå Centre for Global Health Research, Umeå University, Umeå, Sweden

**Keywords:** Childhood, Africa, Asia, mortality, INDEPTH Network, verbal autopsy, InterVA

## Abstract

**Background:**

Childhood mortality, particularly in the first 5 years of life, is a major global concern and the target of Millennium Development Goal 4. Although the majority of childhood deaths occur in Africa and Asia, these are also the regions where such deaths are least likely to be registered. The INDEPTH Network works to alleviate this problem by collating detailed individual data from defined Health and Demographic Surveillance sites. By registering deaths and carrying out verbal autopsies to determine cause of death across many such sites, using standardised methods, the Network seeks to generate population-based mortality statistics that are not otherwise available.

**Objective:**

To present a description of cause-specific mortality rates and fractions over the first 15 years of life as documented by INDEPTH Network sites in sub-Saharan Africa and south-east Asia.

**Design:**

All childhood deaths at INDEPTH sites are routinely registered and followed up with verbal autopsy (VA) interviews. For this study, VA archives were transformed into the WHO 2012 VA standard format and processed using the InterVA-4 model to assign cause of death. Routine surveillance data also provided person-time denominators for mortality rates. Cause-specific mortality rates and cause-specific mortality fractions are presented according to WHO 2012 VA cause groups for neonatal, infant, 1–4 year and 5–14 year age groups.

**Results:**

A total of 28,751 childhood deaths were documented during 4,387,824 person-years over 18 sites. Infant mortality ranged from 11 to 78 per 1,000 live births, with under-5 mortality from 15 to 152 per 1,000 live births. Sites in Vietnam and Kenya accounted for the lowest and highest mortality rates reported.

**Conclusions:**

Many children continue to die from relatively preventable causes, particularly in areas with high rates of malaria and HIV/AIDS. Neonatal mortality persists at relatively high, and perhaps sometimes under-documented, rates. External causes of death are a significant childhood problem in some settings.

Mortality in childhood, particularly in the first 5 years of life, has been a major global concern in recent years. Additional attention has been given to rates of all-cause mortality reduction within the framework of Millennium Development Goal 4 (1) and considerable successes are being achieved in various countries. At the same time, some components of child mortality, for example, deaths in the early days of life, are proving less transigent. Overall burdens of childhood mortality can only be clearly understood when causes of death are reliably attributed, and it has to be recognised that some causes may be more susceptible to reduction than others. At the same time, the mix of causes varies considerably between different settings, as well as between age groups.

Cause-specific childhood mortality in low- and middle-income countries is estimated from a range of sources, including the Child Epidemiology Reference Group (CHERG) (2), and the Global Burden of Disease study (3). However, the data underlying these estimates are often sparse and inconsistent, particularly when it comes to understanding mortality patterns on a population basis (4).

The INDEPTH Network Health and Demographic Surveillance Sites (HDSS) follow vital events within defined populations continuously, and so they provide a means for documenting mortality on a population-related basis (5). Furthermore, by undertaking standardised verbal autopsy (VA) enquiries to follow-up deaths, cause-specific mortality can be assessed within specific childhood age groups to see which cause groups account for substantial components of overall mortality (6).

Our aim in this paper is to describe childhood cause-specific mortality patterns on the basis of a dataset collected at 22 INDEPTH Network HDSSs across Africa and Asia (7). We have chosen here to take as ‘childhood’ the overall age range from birth to 15 years, to give a complete picture of mortality patterns up to adulthood, at the same time providing results separately for the neonatal period, infancy and the under-5 year age group. Although these INDEPTH sites are not constituted as a representative sample, they provide point estimates over a wide range of settings and time periods.

## Methods

The overall INDEPTH dataset (8) from which these childhood mortality analyses are drawn is described in detail elsewhere (7). The Karonga, Malawi, site did not contribute VAs for childhood deaths, and the Purworejo, Indonesia; Farafenni, The Gambia; and Vadu, India, sites carried out verbal autopsies for less than half of the childhood deaths that occurred and/or did not report for the period 2006–2012. Therefore these sites are not considered further here. This leaves documentation on 28,751 deaths in 4,387,824 person-years of observation across 18 sites. VA interviews were successfully completed on 25,357 (88.2%) of the deaths that occurred. A summary of the detailed methods used in common for this series of multisite papers is shown in Box 1.

*Box 1*. Summary of methodology based on the detailed description in the introductory paper (7).

**Age–sex–time standardisation**

To avoid effects of differences and changes in age–sex structures of populations, mortality fractions and rates have been adjusted using the INDEPTH 2013 population standard (9). A weighting factor was calculated for each site, age group, sex and year category in relation to the standard for the corresponding age group and sex, and incorporated into the overall dataset. This is referred to in this paper as age–sex–time standardisation in the contexts where it is used.

**Cause of death assignment**

The InterVA-4 (version 4.02) probabilistic model was used for all the cause of death assignments in the overall dataset (10). InterVA-4 is fully compliant with the WHO 2012 Verbal Autopsy standard and generates causes of death categorised by ICD-10 groups (11). The data reported here were collected before the WHO 2012 VA standard was available, but were transformed into the WHO 2012 and InterVA-4 format to optimise cross-site standardisation in cause of death attribution. For a small proportion of deaths, VA interviews were not successfully completed; a few others contained inadequate information to arrive at a cause of death. InterVA-4 assigns causes of death (maximum 3) with associated likelihoods; thus cases for which likely causes did not total 100% were also assigned a residual indeterminate component. This served as a means of encapsulating uncertainty in cause of death at the individual level within the overall dataset, as well as accounting for 100% of every death.

**Overall dataset**

The overall public-domain dataset (8) thus contains between one and four records for each death, with the sum of likelihoods for each individual being unity. Each record includes a specific cause of death, its likelihood and its age–sex–time weighting.

In this context, all of these data are secondary datasets derived from primary data collected separately by each participating site. In all cases the primary data collection was covered by site-level ethical approvals relating to on-going health and demographic surveillance in those specific locations. No individual identity or household location data were included in the secondary data and no specific ethical approvals were required for these pooled analyses.

## Results

Over the total of 28,751 deaths during 4,387,824 person-years of observation, 5,213 occurred in the neonatal period (first 28 days of life); 8,967 during the remainder of infancy (from one month up to the first birthday); 10,764 in the 1–4 year age group and 3,807 in the 5–14 year age group. All 18 sites reported mortality during at least part of the period 2006–2012, which comprised 68.8% of overall person-time observed; the period 2000–2005 accounted for a further 25.7%. The most natural way to analyse these longitudinal population data across sites is to calculate site-specific mortality rates per 1,000 person-years, shown in Table 1 by age group, period and site. In the sites that have longer-term data, there are some trends reflecting falling childhood mortality. There are also exceptions, however; at the Agincourt, South Africa site, there are clear indications of mortality rising in the middle period, when the HIV/AIDS epidemic was at its height. For the period 2006–12, the highest rates of neonatal mortality were observed in Asian sites, even though they recorded generally lower mortality rates than many African sites in subsequent age groups.

**Table 1:** Childhood all-cause mortality rates per 1,000 person-years by age group and period for 18 INDEPTH HDSS sites

Age group	0–28 days			1–11 months			1–4 years			5–14 years		

Period	<2000	2000–05	2006–12	<2000	2000–05	2006–12	<2000	2000–05	2006–12	<2000	2000–05	2006–12
Bangladesh: Matlab		389.8	357.6		11.8	10.6		3.2	2.3		0.8	0.6
Bangladesh: Bandarban			171.0			28.6			1.9			1.0
Bangladesh: Chakaria			458.0			16.6			4.0			1.0
Bangladesh: AMK		444.7	326.8		11.9	8.0		3.4	2.7		0.8	0.6
Burkina Faso: Nouna	101.2	142.2	92.9	39.3	42.5	24.4	29.8	19.2	12.7	6.0	2.6	1.6
Burkina Faso: Ouagadougou			136.4			20.8			7.8			1.4
Côte d'Ivoire: Taabo			200.9			32.0			15.2			1.8
Ethiopia: Kilite-Awlaelo			188.0			12.6			2.8			1.1
Ghana: Navrongo		305.5	209.7		43.5	22.0		11.4	8.2		2.2	1.7
Ghana: Dodowa			90.4			8.7			4.7			1.4
India: Ballabgarh			280.0			24.4			4.0			0.8
Kenya: Kilifi			160.0			9.6			2.5			0.8
Kenya: Kisumu		302.6	243.0		111.7	74.2		31.7	22.8		2.7	2.4
Kenya: Nairobi		373.3	319.8		58.0	49.8		8.4	6.4		2.1	1.1
Senegal: Niakhar		210.6	126.9		31.2	16.8		20.5	9.9		3.2	1.5
South Africa: Agincourt	81.0	119.7	154.7	13.5	30.3	30.9	4.4	7.0	5.3	0.7	1.0	1.3
South Africa: Africa Centre		151.1	53.0		49.5	27.6		8.9	4.7		1.7	1.2
Vietnam: FilaBavi			123.3			3.0			1.0			0.4

**Fig. 1:**
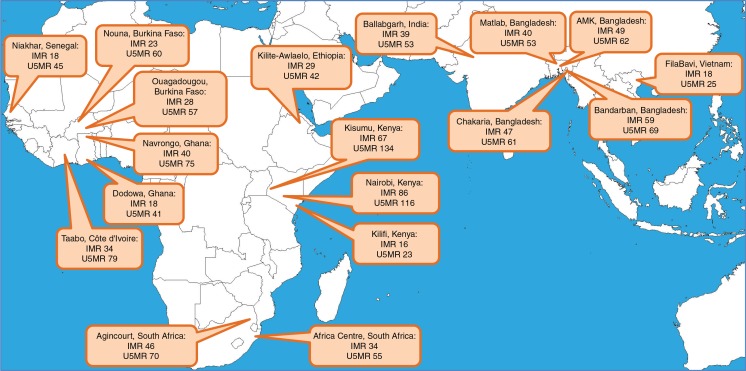
Location of the 18 contributing INDEPTH HDSSs, showing infant mortality rates (deaths in first year of life per 1,000 live births, IMR) and under-5 mortality rates (deaths in first 5 years of life per 1,000 live births, U5MR) for the period 2006–2012.

The terms ‘infant mortality rate’ and ‘under-5 mortality rate’ are frequently used, arguably incorrectly, to refer to numbers of deaths per 1,000 live births, rather than to person-time based rates. However, for the sake of comparability with other sources, Fig. 1 shows these widely used measures of infant and under-5 mortality rates per 1,000 live births for the period 2006–2012, during which all 18 sites reported. The FilaBavi site in Vietnam recorded infant mortality of 11 and under-5 mortality of 15 per 1,000 live births, while the Kisumu site on the northern shores of Lake Victoria recorded infant mortality of 78 and under-5 mortality of 152 per 1,000 live births.

Table 2 shows a detailed breakdown of cause-specific mortality rates per 1,000 person-years by site for major causes and cause groups of childhood mortality. More specific considerations of mortality due to malaria, HIV and external causes are given in accompanying papers (12–14) ; these causes are included here for the sake of completeness rather than for detailed discussion.

**Table 2:** Childhood mortality rates per 1,000 person-years, by cause group and age group, for 18 INDEPTH HDSS sites from 2006 to 2012

Cause	Birth asphyxia	Neonatal infections	Congenital	Prematurity	Diarrhoea	HIV/AIDS	Malaria	Pneumonia	Other infections	External causes	NCDs	Other causes	Indeterminate	All causes
0–28 days														
Bangladesh: Matlab	30.69	116.03	4.67	71.16						0.55		35.77	67.91	326.78
Bangladesh: Bandarban	38.20	73.43										22.58	36.83	171.04
Bangladesh: Chakaria	104.36	42.35	1.97	105.09						1.79		73.27	129.14	457.97
Bangladesh: AMK	53.31	126.26	9.17	25.66								44.75	98.46	357.61
Burkina Faso: Nouna	18.91	44.89	0.63	5.10								2.42	20.93	92.88
Burkina Faso: Ouagadougou	18.42	49.15	7.80	16.90								5.83	38.33	136.43
Côte d'Ivoire: Taabo	53.32	80.20		20.39								15.25	31.75	200.91
Ethiopia: Kilite-Awlaelo	11.56	79.00		4.00								22.12	71.32	188.00
Ghana: Navrongo	52.57	43.80	0.95	57.14								32.36	22.91	209.73
Ghana: Dodowa	11.17	15.45		5.94								16.09	41.79	90.44
India: Ballabgarh	32.37	68.05	2.18	77.48								28.55	71.38	280.01
Kenya: Kilifi	37.98	50.88	3.04	9.92								7.00	51.20	160.02
Kenya: Kisumu	52.66	65.56	2.46	9.61						0.49		27.37	84.88	243.03
Kenya: Nairobi	77.53	80.27		19.41						1.31		37.69	103.61	319.82
Senegal: Niakhar	5.83	51.38	1.10	6.48								13.01	49.11	126.91
South Africa: Agincourt	26.50	73.77		10.99						1.04		13.94	28.40	154.64
South Africa: Africa Centre	10.26	20.89	4.70	1.06						2.47		0.91	12.72	53.01
Vietnam: FilaBavi	31.56			38.58								2.36	50.74	123.24
1–11 months														
Bangladesh: Matlab			0.03		0.30	0.04		4.85	0.92	0.19	0.19	0.86	0.62	8.00
Bangladesh: Bandarban					1.61	1.03	0.85	8.15	0.42		0.77	0.36	15.40	28.59
Bangladesh: Chakaria			1.39		1.55	0.07	0.08	3.46	1.86	0.76	0.59	0.38	6.44	16.58
Bangladesh: AMK			0.05		1.11			7.24	0.48	0.35	0.21	0.43	0.69	10.56
Burkina Faso: Nouna					1.17	0.09	14.14	3.32	0.43	0.59	0.17	0.08	4.40	24.39
Burkina Faso: Ouagadougou			0.64		1.72	1.80	3.03	6.66	0.92	0.15	0.68	0.62	4.64	20.86
Côte d'Ivoire: Taabo			0.96		1.84	2.79	5.83	8.13	2.01		0.78	0.62	9.04	32.00
Ethiopia: Kilite-Awlaelo			0.31			0.65	0.32	6.14	0.09			0.09	5.00	12.60
Ghana: Navrongo			0.69		3.14	0.99	2.59	4.93	2.09	0.44	1.11	0.25	5.76	21.99
Ghana: Dodowa					0.51	0.39	0.36	3.47	0.23	0.15	0.16	0.35	3.12	8.74
India: Ballabgarh			0.63		4.16		0.71	8.88	0.99	0.53	1.20	0.39	6.94	24.43
Kenya: Kilifi			0.12		0.40	1.94	1.08	2.48	0.60	0.08	0.19	0.08	2.60	9.57
Kenya: Kisumu			0.34		6.55	7.54	17.99	25.49	2.74	0.49	1.70	0.44	10.96	74.24
Kenya: Nairobi			0.04		2.90	3.84	1.16	16.71	6.48	0.99	0.17	0.23	17.26	49.78
Senegal: Niakhar					6.10	0.10	1.86	2.03	0.55	0.06	0.86		5.21	16.77
South Africa: Agincourt			0.23		3.32	4.42	0.75	12.90	2.79	0.26	0.55	0.21	5.47	30.90
South Africa: Africa Centre			0.29		1.87	3.97	0.26	15.96	0.59	0.22	0.24	0.49	3.65	27.54
Vietnam: FilaBavi						0.16		1.82	0.46				0.57	3.01
1–4 years														
Bangladesh: Matlab			0.01		0.03	0.03	0.00	0.44	0.15	1.11	0.04	0.62	0.22	2.65
Bangladesh: Bandarban					0.17		0.17	0.16	0.17	0.35	0.29		0.59	1.90
Bangladesh: Chakaria					0.27		0.06	0.78	0.27	1.34	0.28	0.04	0.92	3.96
Bangladesh: AMK					0.27	0.03		0.49	0.05	1.37		0.01	0.03	2.25
Burkina Faso: Nouna					0.98	0.12	6.91	1.46	0.16	0.28	0.10	0.08	2.64	12.73
Burkina Faso: Ouagadougou			0.03		0.48	0.58	2.43	1.03	0.39	0.11	0.29	0.64	1.78	7.76
Côte d'Ivoire: Taabo			0.07		0.70	1.55	4.88	1.50	0.54	0.23	0.58	0.24	4.92	15.21
Ethiopia: Kilite-Awlaelo					0.15	0.22	0.17	0.30	0.12	0.11	0.22	0.06	1.49	2.84
Ghana: Navrongo			0.05		0.74	0.76	2.08	0.53	0.46	0.44	0.83	0.24	2.04	8.17
Ghana: Dodowa					0.13	0.19	0.85	0.98	0.18	0.17	0.21	0.14	1.87	4.72
India: Ballabgarh			0.03		0.72	0.05	0.59	0.61	0.07	0.42	0.12	0.06	1.30	3.97
Kenya: Kilifi			0.01		0.09	0.48	0.61	0.33	0.07	0.09	0.08	0.06	0.70	2.52
Kenya: Kisumu					1.26	5.18	7.61	2.46	0.60	0.41	0.75	0.71	3.82	22.80
Kenya: Nairobi					0.35	1.04	0.22	0.95	1.18	0.37	0.04	0.10	2.18	6.43
Senegal: Niakhar					2.94	0.24	3.45	0.38	0.15		0.72	0.05	1.96	9.89
South Africa: Agincourt			0.02		0.34	1.88	0.28	1.07	0.43	0.17	0.23	0.15	0.78	5.35
South Africa: Africa Centre			0.07		0.10	1.25	0.12	1.46	0.25	0.39	0.06	0.13	0.88	4.71
Vietnam: FilaBavi			0.09					0.29	0.08			0.16	0.34	0.96
5–14 years														
Bangladesh: Matlab						0.00		0.05	0.09	0.19	0.09	0.06	0.07	0.55
Bangladesh: Bandarban						0.07	0.18	0.07	0.07	0.15	0.10	0.06	0.33	1.03
Bangladesh: Chakaria							0.03	0.04	0.15	0.34	0.17	0.02	0.25	1.00
Bangladesh: AMK						0.01		0.07	0.09	0.19	0.12	0.01	0.07	0.56
Burkina Faso: Nouna						0.02	0.60	0.14	0.07	0.11	0.20		0.49	1.63
Burkina Faso: Ouagadougou						0.09	0.35	0.16	0.10	0.13	0.05	0.06	0.41	1.35
Côte d'Ivoire: Taabo						0.27	0.27	0.25	0.11	0.22	0.12	0.06	0.48	1.78
Ethiopia: Kilite-Awlaelo						0.02	0.03	0.05	0.07	0.28	0.11	0.04	0.45	1.05
Ghana: Navrongo						0.07	0.13	0.08	0.17	0.40	0.46	0.02	0.41	1.74
Ghana: Dodowa						0.07	0.21	0.20	0.13	0.14	0.11	0.01	0.46	1.33
India: Ballabgarh						0.01	0.05	0.01	0.09	0.22	0.10	0.01	0.35	0.84
Kenya: Kilifi						0.20	0.12	0.07	0.06	0.13	0.05	0.00	0.15	0.78
Kenya: Kisumu						0.44	0.59	0.30	0.18	0.14	0.23	0.04	0.47	2.39
Kenya: Nairobi						0.11	0.05	0.05	0.24	0.15	0.16	0.00	0.31	1.07
Senegal: Niakhar						0.16	0.30	0.08	0.12		0.30	0.01	0.53	1.50
South Africa: Agincourt						0.28	0.05	0.23	0.24	0.11	0.10	0.01	0.32	1.34
South Africa: Africa Centre						0.16	0.01	0.07	0.41	0.25	0.15	0.01	0.16	1.22
Vietnam: FilaBavi									0.07		0.04		0.25	0.36

**Fig. 2:**
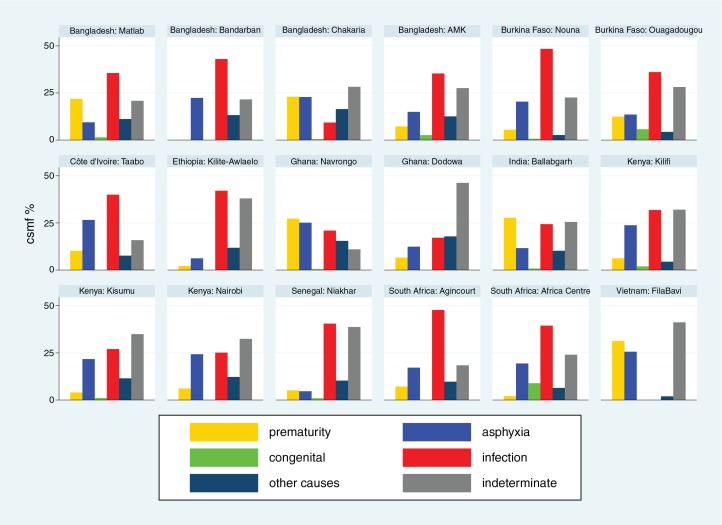
Cause-specific mortality fractions (CSMF) for major cause of death groups for neonates at 18 INDEPTH sites during 2006–2012.

**Fig. 3:**
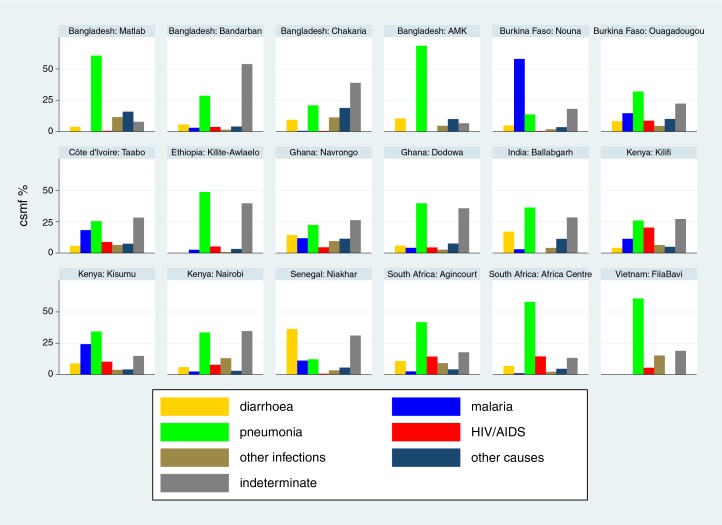
Cause-specific mortality fractions (CSMF) for major cause of death groups for infants (1–11 months) at 18 INDEPTH sites during 2006–2012.

**Fig. 4:**
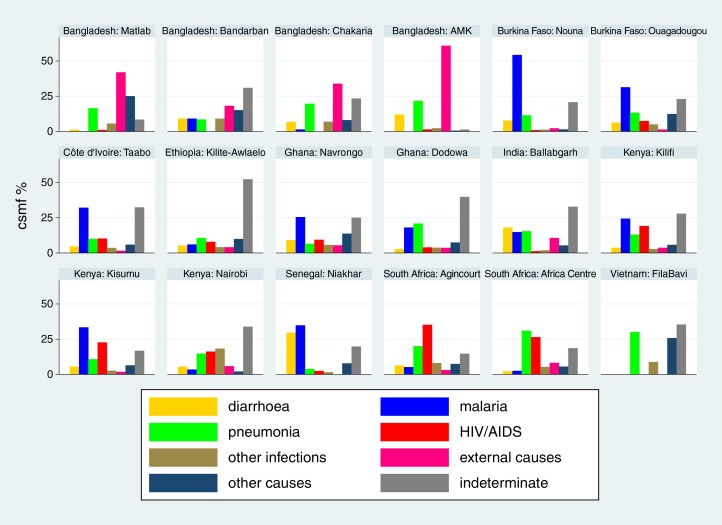
Cause-specific mortality fractions (CSMF) for major cause of death groups for children aged 1–4 years at 18 INDEPTH sites during 2006–2012.

**Fig. 5:**
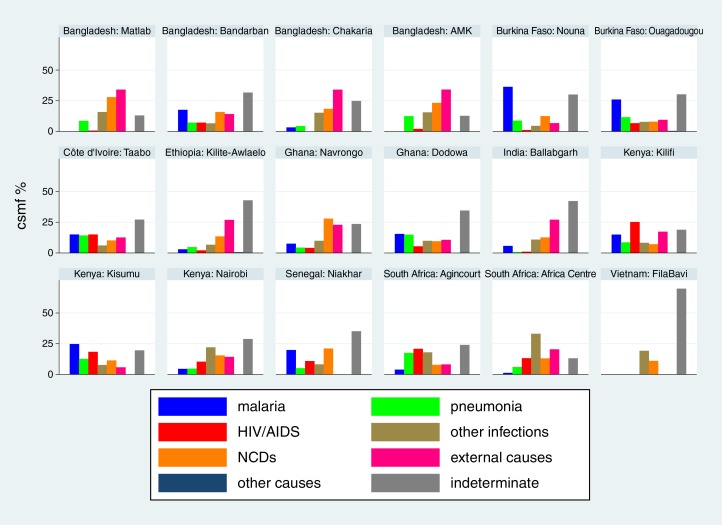
Cause-specific mortality fractions (CSMF) for major cause of death groups for children aged 5–14 years at 18 INDEPTH sites during 2006–2012.

Cause-specific mortality fractions (CSMF) for each of the 18 sites (15–32) are shown separately for each age group (neonates, 1–11 months, 1–4 years and 5–14 years) in Figs. 2–5 respectively, to give a sense of what the dominant causes of mortality are in particular sites and age groups. For most sites, infections accounted for the largest proportion of neonatal deaths, although prematurity was also an important cause in some settings. Pneumonia dominated as the major cause of infant deaths, although malaria was also important in some endemic areas. Local factors dictated major causes in the 1–4 year age group, from external causes in Bangladesh to malaria and HIV/AIDS in highly endemic settings. For the 5–14 year age group, external causes and, in some places, malaria continued as important causes, while there was also an increased proportion of mortality due to non-communicable diseases in some sites.

## Discussion

This large-scale description of childhood mortality, all based on individually documented deaths within defined populations in Africa and Asia, provides important insights into the continuing causes of deaths that would be regarded as largely preventable in other parts of the world. Despite encouraging progress in reducing childhood mortality in many places, the data presented here reflect an overall situation from these sites in recent years where around 1 out of every 12 children born does not survive into adulthood.

The causes behind these individual tragedies are multifactorial, including poverty, living conditions and health services, and cannot be explored in full detail from the data presented here. What we do know from these cause of death data is that there are substantial differences in patterns of childhood mortality between countries, and in some cases, such as Kenya, also widespread within-country variation. Certain infectious diseases, particularly malaria and HIV/AIDS, contribute major components of childhood mortality in settings where they occur commonly, and hence account for a substantial part of variation in overall mortality. For example, at the Kisumu, Kenya, site malaria and HIV/AIDS together accounted for 56% of deaths in the 1–4 year age group; but other causes did not occur at rates that were markedly different from those in a number of other sites. Conversely, at the FilaBavi, Vietnam site, where overall mortality in the 1–4 year age group was less than 10% of the level observed in Kisumu, pneumonia accounted for 29% of the relatively few deaths that did occur. Similarly, it is very obvious from Fig. 4 that external causes of death are a major problem in Bangladesh. This illustrates the importance of considering both population-based cause-specific mortality rates and CSMF when coming to any understanding of significant causes of mortality burdens within particular populations. Both parameters are essential information to have when considering interventions, either for specific diseases or for health promotion in general.

One of the strengths of maintaining surveillance of all deaths within particular populations, as the INDEPTH HDSS sites do, is that by definition the results for each particular cause constitute a clear component of 100% total mortality. By contrast, when deaths due to particular causes are documented at health facilities, or in vertical disease-oriented programmes, denominators are always unclear. In addition, the InterVA-4 methodology that was used in this dataset captures the uncertainty around cause of death assignment at the individual level, which is presented here as part of the ‘indeterminate’ category. Although some studies have sought to reclassify so-called ‘garbage’ cause of death codes into more specific groupings (33), in reality the assignment of cause of death at the individual level is not something that can proceed with total certainty for every case, irrespective of the methods used, and this may be particularly true for some childhood deaths. We contend therefore that maintaining an ‘indeterminate’ category that encompasses uncertainty in individual cause of death assignments, as well as accounting for a minority of deaths for which a VA interview was for some reason not possible, is an important and realistic concept in these population-based analyses (34). Whether or not the ‘indeterminate’ group actually constitutes a similar mix of causes of death as those that are successfully assigned has to remain a matter for conjecture.

Because childhood mortality globally is reported as falling, we have concentrated our analyses here on data from the 2006 to 2012 period to reflect a relatively contemporary scenario. A detailed comparative study by country, age group and cause of death between these results and other findings on cause-specific child mortality is beyond the scope of this paper. However, some selected comparisons can be made. The GBD2010 global estimates of child mortality (35) are approximately contemporaneous with the 2006–12 time period presented here, as are the 2010 mortality estimates presented by UNICEF in the State of the World's Children 2012 (36). The basic rates of all-cause childhood mortality are reasonably congruent between these sources and the findings from the INDEPTH sites, although this is by no means a precise comparison (specific population site measurements versus national estimates). The perennial concern that some early neonatal deaths may have been considered as stillbirths (37), and therefore not registered as deaths, may have been an issue at some of the INDEPTH sites in Africa that registered fairly low neonatal mortality rates in comparison to infant mortality. More systematic application of the WHO 2012 VA tool (11) in the future may help to resolve this, since it contains a number of questions specifically aimed at making this distinction. Operationally, it is probably important to consider undertaking a VA interview for all third trimester pregnancies that do not result in a live baby, rather than making *a priori* distinctions between stillbirths and early neonatal deaths, in order to capture all available information. Local cultural and spiritual beliefs around the deaths of babies may also be an important consideration.

## Conclusions

Individual deaths in childhood are always causes for great sadness; all the more so if the circumstances and the eventual cause of death mean that survival could have been reasonably possible. These analyses of individual deaths show that large numbers of children in Africa and Asia continue to die of avoidable causes, starting from suboptimal delivery care, through treatable infections and preventable accidents. Despite some countries achieving MDG4 targets, there is still room for further improvement. Documenting the magnitude of the various leading causes of childhood death, across relevant age groups, is a pre-requisite for planning effective survival interventions.
